# The *Brucella* TIR domain containing proteins BtpA and BtpB have a structural WxxxE motif important for protection against microtubule depolymerisation

**DOI:** 10.1186/s12964-014-0053-y

**Published:** 2014-10-12

**Authors:** Christine Felix, Burcu Kaplan Türköz, Sebastien Ranaldi, Thomas Koelblen, Laurent Terradot, David O’Callaghan, Annette Caroline Vergunst

**Affiliations:** INSERM, U1047, 186 Chemin du Carreau de Lanes, 30908 Nîmes, Cedex 02, France; INSERM U1047, Université Montpellier 1, UFR Médecine, 186 Chemin du Carreau de Lanes, 30908 Nîmes, Cedex 02, France; UMR 5086, BMSSI, CNRS- Université Lyon 1, Institut de Biologie et Chimie des Protéines, 7 Passage du Vercors, Lyon, F-69367 France; Current address: Ege University, Faculty of Engineering, Department of Food Engineering, 35100 Bornova, Izmir Turkey

**Keywords:** BtpA, TcpB, BtpB, Brucella, Bacterial TIR domain proteins, TIR domain, WxxxE motif, GTPase signalling, Microtubule dynamics

## Abstract

**Background:**

The TIR domain-containing proteins BtpA/Btp1/TcpB and BtpB are translocated into host cells by the facultative intracellular bacterial pathogen *Brucella.* Here, they interfere with Toll like receptor signalling to temper the host inflammatory response. BtpA has also been found to modulate microtubule dynamics. In both proteins we identified a WxxxE motif, previously shown to be an essential structural component in a family of bacterial type III secretion system effectors that modulate host actin dynamics by functioning as guanine nucleotide exchange factors of host GTPases. We analysed a role for the WxxxE motif in association of BtpA and BtpB with the cytoskeleton.

**Results:**

Unlike BtpA, ectopically expressed BtpB did not show a tubular localisation, but was found ubiquitously in the cytoplasm and the nucleus, and often appeared in discrete punctae in HeLa cells. BtpB was able to protect microtubules from drug-induced destabilisation similar to BtpA. The WxxxE motif was important for the ability of BtpA and BtpB to protect microtubules against destabilising drugs. Surprisingly, ectopic expression of BtpA, although not BtpB, in HeLa cells induced the formation of filopodia. This process was invariably dependent of the WxxxE motif. Our recent resolution of the crystal structure of the BtpA TIR domain reveals that the motif positions a glycine residue that has previously been shown to be essential for interaction of BtpA with microtubules.

**Conclusions:**

Our results suggest a structural role for the WxxxE motif in the association of BtpA and BtpB with microtubules, as with the WxxxE GEF family proteins where the motif positions an adjacent catalytic loop important for interaction with specific Rho GTPases. In addition, the ability of ectopically expressed BtpA to induce filopodia in a WxxxE-dependent manner suggests a novel property for BtpA. A conserved WxxxE motif is found in most bacterial and several eukaryotic TIR domain proteins. Despite the similarity between ectopically expressed BtpA and WxxxE GEFs to modulate host actin dynamics, our results suggest that BtpA is not part of this WxxxE GEF family. The WxxxE motif may therefore be a more common structural motif than thus far described. BtpA may provide clues to cross-talk between the TLR and GTPase signalling pathways.

**Electronic supplementary material:**

The online version of this article (doi:10.1186/s12964-014-0053-y) contains supplementary material, which is available to authorized users.

## Background

*Brucella* is a Gram negative facultative intracellular bacterium that can cause brucellosis or Malta Fever, the world’s most widespread zoonotic disease [1]. In humans *Brucella* causes an undulant fever that can be accompanied by complications such as endocarditis, arthritis and osteomyelitis [2]. *Brucella* is a stealth pathogen, known for its silent entry into host cells and multiple mechanisms to suppress host innate immune signalling, including non-toxic lipid A, and avoidance of oxidative burst and Toll like receptor (TLR) signalling cascades [2-6]. Whereas the primary task of immune cells is to phagocytose and degrade microbes, many intracellular pathogens, including *Brucella*, use secretion systems to introduce bacterial effector proteins directly into host cells to alter host cell biology and favour their intracellular replication [7,8]. For full virulence, *Brucella* requires its VirB type IV secretion system (T4SS) to modulate endosomal trafficking, and create a replication niche in ER-derived membrane vesicles, named *Brucella* containing vacuoles (BCV) [9-11]. For many years, the effector proteins translocated by the *Brucella* VirB system remained elusive. After the identification of a first candidate [12], recent efforts from several laboratories, using both bioinformatics screens and translocation assays have resulted in a list of possible effectors [13-16]. To date, the precise role of most of these proteins in *Brucella* virulence is still not clear and the subject of intense research.

Recently, the proteins BtpA (Btp1/TcpB) and BtpB have been shown to be translocated by *Brucella* into host cells [17]. Although a TEM-1 β-lactamase assay did not show a significant difference in translocation efficiency of the proteins from wild type and *virB* mutant bacteria, a CyA reporter assay showed VirB-dependent protein transport into host cells, suggesting BtpA and BtpB may be substrates of the VirB T4SS. BtpA and BtpB belong to a class of bacterial proteins first described in *Salmonella*, *Escherichia coli* and *Brucella* that share homology with the eukaryotic Toll/Interleukin-1 receptor (TIR) domain [18,19]. A conserved TIR domain is present in eukaryotic TLR proteins as well as their downstream signalling TIR adaptor proteins, including the central cytosolic adapter protein MyD88. The TIR domain is essential for TLR and adaptor interactions and for the onset of a signalling cascade resulting in nuclear translocation of the transcription factor NFκB, followed by the production of pro-inflammatory cytokines and type I interferons [20]. TIR domain interactions play a key role in activating conserved cellular signal transduction pathways in response to pathogen signals, and it was suggested that bacterial TIR proteins interfere with host TLR defence signalling by molecular mimicry (reviewed in [21]).

*B. abortus* 2308 BtpA (BAB1_0279) and the almost identical *B. melitensis* 16M BtpA (BMEI1674) down modulate maturation of dendritic cells [22] and inhibit TLR-induced NFκB activation. It has been suggested that this is through interference with the TLR4/MyD88/TIRAP complex [23-27], however the exact binding partner of BtpA is still a subject of controversy. Far less is known about BtpB, however recently it has also been shown to play a role in immune modulation [17]. Recently we, and others, published the crystal structure of the BtpA TIR domain, which showed a dimeric arrangement of a canonical TIR domain [25,28,29], but significant differences in loop structures suggest that these proteins could interfere with intrinsic host TLR signalling pathways. These recent advances will help to more precisely determine the exact molecular mechanisms of this molecular mimicry, and to further address the different roles these proteins may play in virulence.

In addition to an important role for BtpA and BtpB in inhibiting TLR-mediated immune responses, *in vitro* and overexpression studies have shown that BtpA tightly interacts with microtubules and is able to stabilize polymerized microtubules by inhibiting microtubule disassembly induced by the microtubule-drug nocodazole [30]. Here we show that BtpB, in addition to BtpA, is also able to protect microtubules from drug-induced depolymerization. We have identified a WxxxE motif in BtpA and BtpB. A conserved WxxxE motif has been shown to have a structural role in a family of T3SS effectors, which transiently subvert host actin dynamics by functioning as Guanine nucleotide Exchange Factors (GEFs) of Rho GTPases [31,32]. We provide evidence that the WxxxE motif in BtpA and BtpB is an important structural determinant for protection of microtubules suggesting a functional association with microtubules for both proteins. In addition, we show that ectopically expressed BtpA induces the formation of filopodia in a WxxxE-dependent manner. Our results are supported by the crystal structure of BtpA.

## Results

### A conserved WxxxE motif in BtpA organizes functional surface loops

**Figure 1:**
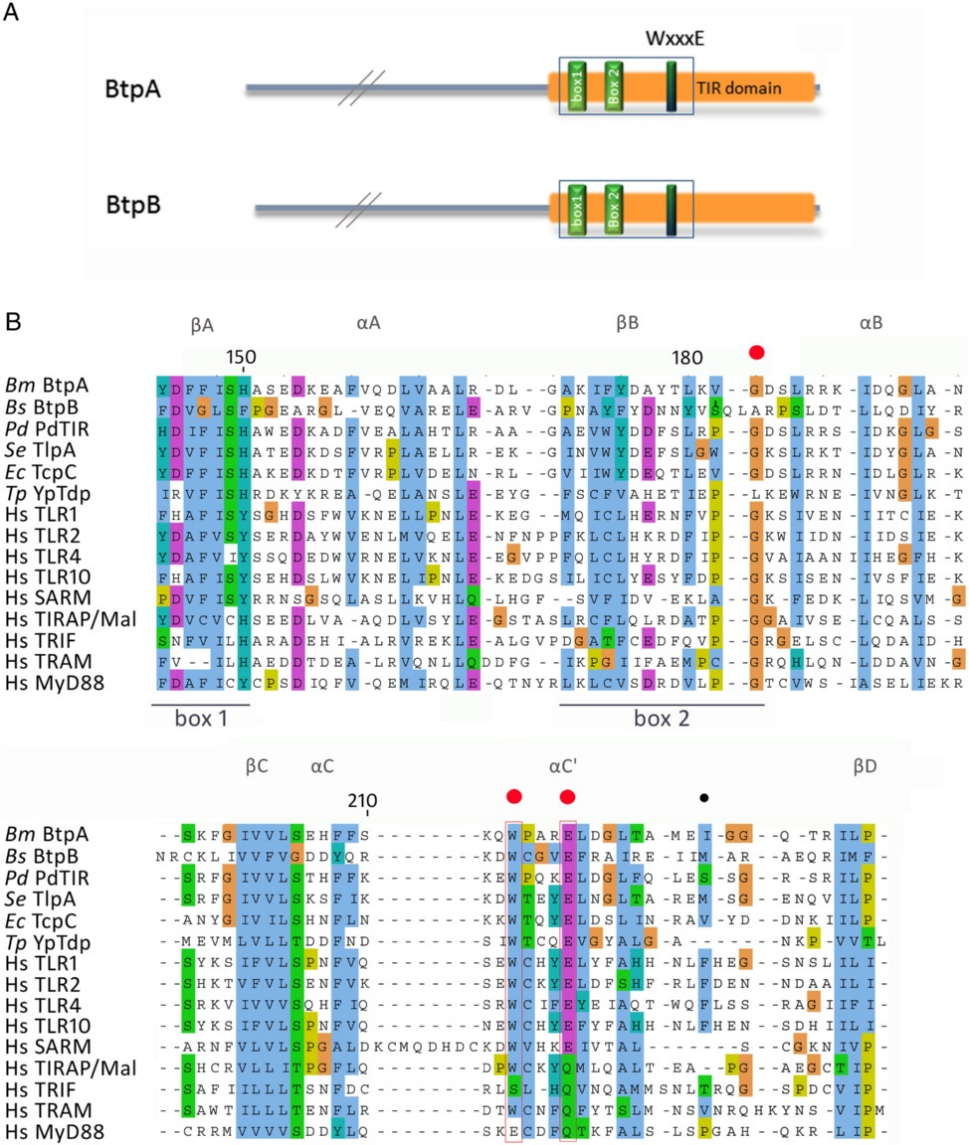
**Btp protein structure, and sequence alignment indicating TIR domains and position of WxxxE motif. (A)** Schematic representation of the *Brucella* BtpA and BtpB proteins with the position of TIR domain boxes and WxxxE motif indicated. **(B)** Multiple sequence alignment of *Brucella melitensis* BtpA (AAL52855.1), *Brucella suis* BtpB (AAN29664.1), *Paracoccus denitrificans* PdTir (3H16_D), *Salmonella enterica* TlpA (WP_000116933.1), *Escherichia coli* TcpC (ADO30450), *Yersinia pestis* YpTdp (NP_669733.1), human TLR1 (AAY85643.1), human TLR2 (AGR82491.1), human TLR4 (AAY82267.1), human TLR10 (AAY78485.1), SARM (AAH40429.1), Mal/TIRAP (AF406652_1), TRIF (AAH09860), TRAM (NP_067681), MyD88 (AAB49967.1). The alignment shows the sequence encompassing the TIR domain boxes 1 and 2, and WxxxE motif. The alignment was performed with T-coffee analysis, and edited using Jalview, using the ClustalX colour scheme. Structural features indicated above the sequence alignment according to Kaplan-Türköz [28]. The residues mutated in this study are indicated with red (W213, E217, and G183) and black (I226) dots.

**Figure 2:**
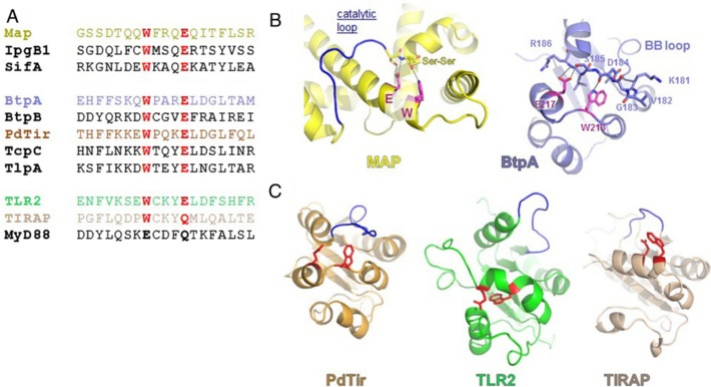
**Conservation of the WxxxE motif. (A)**. Sequence alignment of the WxxxE containing T3SS effectors Map, IpgB1 and SifA; the bacterial TIR proteins BtpA, BtpB, PdTir, TcpC and TlpA; and eukaryotic hTLR2, hTIRAP and hMyD88. **(B)**. Structural comparison of Map (yellow) and BtpA (slate) structure depicted as ribbons and highlighting the residues W and E (purple), and catalytic and BB loops (blue). Side chains are shown in ball and sticks for the residues involved in the structural scaffold (see text for details). **(C)**. Comparison of the WxxxE motif (with W and E residues shown as red balls and sticks) and BB loop (blue) organisation in the structures of PdTir (gold), TLR2 (green) and TIRAP (wheat).

BtpA and BtpB have both been shown to have immune modulatory functions in the host cell through their TIR domains by directly interfering with TLR signalling [17], supported by recent structural information [25,28,29]. *btpA* and *btpB* are located on different genomic islands [33]. We analysed 407 genomes in the PATRIC database (http://patricbrc.vbi.vt.edu/portal/portal/patric/Home, January 2014). Whereas the GI carrying *btpB* is found in the genomes of all the classical *Brucella* strains, the GI containing *btpA* shows variable distribution (Additional file 1: Figure S1). The *btpA* and *btpB* genes show 11% amino acid identity, and are highly conserved throughout the genus. In addition to the well-studied TIR domain, we found that BtpA and BtpB contain a WxxxE motif (Figure 1) (except in all *B. melitensis* which have the Trp residue changed into Arg in BtpB). A conserved WxxxE structural motif has been described to be important in a family of bacterial type III secretion system effectors, including *Shigella* IpgB1 and enteropathogenic *Escherichia coli* Map (Figure 2A), for their function as guanine nucleotide exchange (GEF) mimics for Rho GTPases [31,34].

A large family of TIR proteins in both pathogenic and non-pathogenic bacteria has recently been described [21,35]. Interestingly, sequence alignment shows that the WxxxE motif is highly conserved among the bacterial TIR proteins, including *Paracoccus denitrificans* PdTir, *Yersinia pestis* YpTdp, *Salmonella enterica* TlpA, and *Escherichia coli* TcpC (Figures 1B, 2A) and eukaryotic TLR proteins (TLR1, 2, 4, 6 and 10). Although the motif is not conserved in the TIR adaptor proteins TIRAP/Mal, TRAM, MyD88 and TRIF, it is present in SARM (Figure 1B), the only TIR adaptor protein that has been shown to negatively regulate TLR signalling [36]. The conservation of the WxxxE motif in a large number of TIR domain proteins suggests the WxxxE motif could have an important structural role, as shown for the WxxxE GEF family proteins.

In the bacterial GEF proteins, the WxxxE motif has been shown to be instrumental in positioning the so-called catalytic loop that participates in the interaction with GTPases such as Cdc42 or Rac [37] (Figure 2A). In the BtpA structure, the WxxxE motif is located in the αC helix (Figure 1B) and interacts with the C-terminal part of the BB loop (Figure 2B). The nitrogen of the tryptophan ring interacts with the hydroxyl group of the D184 main chain while the side chain of E217 interacts with the S185 (Figure 2B). This organization is reminiscent of the WxxxE bonding with the SS motif of the catalytic loop in the EPEC Map effector ([34], Figure 2B). In BtpA, however, this interaction positions a glycine residue (G183) in close proximity to the WxxxE pocket (Figure 2B). Interestingly, G183 has been shown to be important for interaction of BtpA with microtubules (G158 in [23,24]). Other than the WxxxE motif, no significant structural sequence similarity is present between known WxxxE family GEFs and BtpA. It is of note that the *Brucella* BtpB protein does not have the G residue at the corresponding position.

Despite the high degree of conservation of the WxxxE motif in the family of bacterial and eukaryotic TIR proteins, structural comparison between the available structures indicates that the interaction between the WxxxE motif and the D residue in the BB loop only occurs in BtpA and PdTIR, and not any of the eukaryotic TIR proteins (Figure 2C). In TLR2 for instance, the WxxxE packs against the DD loop and αD helix (Figure 2C). Together, these observations indicate that a conserved WxxxE motif may be an important structural component in a family of bacterial TIR proteins by positioning different loop structures. More specifically, in BtpA the WxxxE motif may play a structural role by positioning the G183 residue for interaction with microtubules.

### The WxxxE motif is important for subcellular localisation of BtpA and protection of microtubules

**Figure 3:**
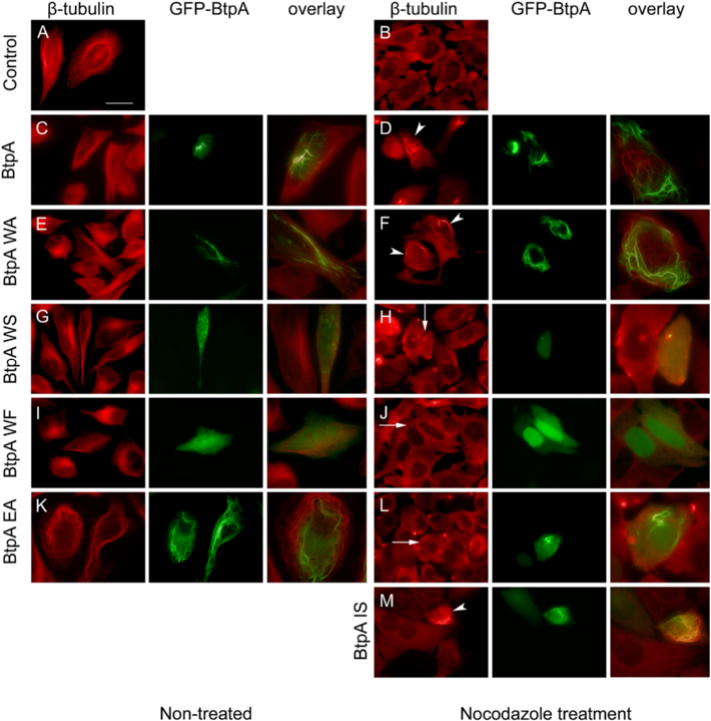
**Interaction with and stabilization of microtubules by BtpA against Nocodazole-induced depolymerization is WxxxE-dependent.** HeLa cells were transfected with plasmids expressing GFP-BtpA **(C, D)**, GFP-BtpA WxxxE variants **(E-L)**, or I226S **(M)**, respectively. Sixteen hours after transfection cells were either left untreated **(A, C, E, G, I, K)** or treated with 1 μg/ml nocodazole for 30 min **(B, D, F, H, J, L, M)**. Nocodazole-treated and control cells were fixed and stained with mouse anti β-tubulin antibody, followed by detection with anti-mouse Texas Red, and analysed by fluorescence microscopy. **(A)** Fluorescent image of tubulin-stained non-transfected control cells **(B)** Fluorescent image of non-transfected, nocodazole-treated cells. **(C-M)** Individual green and red fluorescent images and overlay image of HeLa cells expressing **(C-D)** GFP-BtpA, **(E-F)** GFP-BtpA W213A, **(G-H)** GFP-BtpA W213S, **(I-J)** GFP-BtpA W213F, **(K-L)** GFP-BtpA E217A, **(M)** GFP-BtpA I226S. Arrow heads indicate individual cells showing inhibition of nocodazole-induced microtubule depolymerization by expression of **(D)** GFP-BtpA, **(F)** GFP-BtpA W213A, and **(M)** and I226S, in contrast to non-transfected cells. Arrows indicate nocodazole-induced microtubule depolymerization in individual cells expressing **(H)** GFP-BtpA W213S, **(J)** GFP-BtpA W213F, **(L)** GFP-BtpA E217A. Scale bar, 25 μm indicated in A applies to all individual green and red fluorescent images. The overlay images are close ups of individual cells in the corresponding red and green fluorescent images. Data are representative of 3 or more independent experiments.

Besides its important role in interference with TLR signalling BtpA has been shown to modulate microtubule dynamics and protect microtubules from drug-induced depolymerisation [30]. To address a possible role for the WxxxE motif in interaction of BtpA with microtubules, we performed ectopic expression analysis to study the subcellular localisation of the wild type protein and a panel of variants. We created N-terminal fusions of BtpA to GFP in a eukaryotic expression plasmid, and transfected HeLa cells. Confocal and fluorescence microscopy showed that GFP-BtpA formed a filamentous network in the cell (Additional file 2: Figure S2, and Figure 3C, and [24]). In contrast to Radhakrishnan et al. [24], we did not find any evidence for rounding up or shrinkage of cells. Importantly, localisation of BtpA was similar when expressed without the GFP tag and detected with anti-BtpA antibodies (data not shown).

GFP-BtpA predominantly colocalised with microtubules, labelled with anti-β-tubulin antibodies (Additional file 3: Figure S3, and [24]). Confocal imaging clearly showed that the BtpA tubules localised at the periphery of the cell and, although not colocalising along the complete microtubule network, they showed a regular interaction with the cytoskeleton. Similar to observations reported by Radhakrishnan and colleagues, BtpA could prevent the microtubule destabilizing effect of nocodazole (Figure 3C, D).

Next, we analysed the subcellular localisation of different GFP-BtpA WxxxE variants and their effect on microtubule destabilisation by nocodazole. Using site-directed mutagenesis with the GFP-BtpA expression plasmid as a template, the substitutions W213A, W213F, W213S, E217D, E217A, as well as I226S, and G183A were introduced. Despite very low amino acid sequence homology between BtpA and BtpB, and members of the WxxxE GEF family, we identified a conserved isoleucine residue, 9 amino acids downstream of the WxxxE motif and included a variant in our study. Subcellular localisation of the GFP-fusion proteins was confirmed using BtpA antibodies (data not shown). The different GFP-BtpA variants were ectopically expressed in HeLa cells and details are presented in Table 1 and Figure 3. Although the W213A variant behaved as wild type BtpA, both in subcellular localisation as well as its ability to prevent microtubule destabilisation (Figure 3E, F), substitution of W213 with F or S resulted in a diffuse GFP signal in the cell, indicating these changes greatly affected the localisation of GFP-BtpA. Not localising with microtubules was correlated with the inability of these variant proteins to protect microtubules from degradation by nocodazole (Figure 3G-J); interestingly, in 1.9% of the transfected cells the W213F variant localised in a tubular structure and, in these cells, was able to prevent degradation of microtubules, showing the substitution did not affect the ability of the W213F variant to protect microtubules from depolymerisation when localised correctly.

**Table 1:** **Influence of mutations in the WxxxE motif of BtpA and BtpB on subcellular localisation**, **microtubule**-**protection and filopodia induction**

**BtpA or variant**	**Localisation of GFP-** **BtpA in filaments (% of transfected cells ± ** **SD)**	**Protection of microtubules after NC treatment** ** (% of cells with BtpA arranged in filaments)** ^**a**^	**Induction of filopodia** **(% of cells with BtpA arranged in filaments)** ^**b**^
BtpA	90.5 ± 1.36	73,8 ± 3.45	75.9 ± 0.26
BtpA W213S	0	0	0
BtpA W213A	90.1 ± 3.86	75,6 ± 5,00	0
BtpA W213F	81.9 ± 1.81	87,1 ± 5,36	0
BtpA E217D	1.3 ± 0.78	83.9 ± 9.89	0
BtpA E217A	52.6 ± 6.67	0	0
BtpA I226S	14.4 ± 1.93	76,1 ± 3,66	69.7 ± 3.5
BtpA G183A	0	0	0
**BtpB or variant**	**Protection of microtubules after NC treatment (% of cells with)** ^**c**^		**Induction of filopodia**
	**Diffuse BtpB pattern**	**Punctate BtpB pattern**	
BtpB	24.5 ± 0.71	82,2 ± 1.59	0
BtpB W263S	0	0	0
BtpB E267A	0	0	0

HeLa cells were transfected with plasmid DNA harbouring pCMV:GFP-BtpA, pCMV:GFP-BtpB or the respective Btp variants. After 16–20 hours cells were left untreated or treated with 1 μg/ml nocodazole for 30 min and fixed. Microtubules were visualised using β-tubulin antibodies and detected with Texas-Red conjugated secondary antibodies. For localisation studies, at least 200 transfected cells were analysed per variant per experiment. Data are given in average percentage of transfected cells ± SD (of three independent experiments per variant). For analysis of filopodia, cells were fixed 16-20h hours after transfection, and actin detected with Rhodamine Phalloidin. Each variant was analysed in at least 3 independent experiments.

^a^For wildtype BtpA and variants, protection of nocodazole-induced microtubule destabilisation was observed only in transfected cells showing BtpA in filaments. Indicated is the average percentage of cells with filaments that show protection, calculated from two individual experiments (±SD). Cells that showed partial protection were not taken into consideration. At least 100 cells per experiment were counted for BtpA wildtype, BtpA W213A, and BtpAI226S. For BtpA W213F and BtpA E217D, seen the low percentage of transfected cells with BtpA localising in filaments, about 12 cells with BtpA organised in filaments were counted in 2 independent experiments. BtpA W213S, BtpAE217A and BtpAG183A did not show any protection.

^b^Wildtype BtpA and the I226S variant showed induction of filopodia only in transfected cells showing BtpA in filaments. Counts are based on a total of 67 cells with BtpA in filaments in each of 2 independent experiments.

^c^BtpB transfected cells showed a large variation in ectopic expression phenotypes: Low GFP expression, high GFP expression, and with or without punctae (varying from 1 to over 50). As for BtpA, cells showing partial protection were not taken into account. In two independent experiments 62 and 48 cells (of which 32.7% ± 10.8 were cells without punctae), respectively were analysed for protection of microtubules.

The E217A variant, however, was still found in tubular structures in approximately 53% of the transfected cells yet, in contrast to the W213A and W213F variants, was unable to prevent degradation of microtubules by nocodazole even in those cells (Figure 3K, L). Interestingly, a conservative substitution resulting in the E217D variant, greatly affected GFP-BtpA localisation (Table 1), but again, in about 1% of the transfected cells the E217D variant localised in a tubular structure and in these cells was able to prevent degradation of microtubules. These results demonstrate that the Glu residue is also important for localisation of GFP-BtpA, and suggest in addition that negative charge at this position is essential for the ability of BtpA to protect microtubules from degradation.

The G183 residue lays adjacent to the WxxxE motif in the BtpA crystal structure (Figure 2B). The G183A variant behaved as reported earlier [24] and was unable to colocalise with microtubules, and expectedly unable to prevent microtubule degradation (not shown). The I226S variant formed a tubular network in about 14% of the cells (Table 1), and was able to prevent microtubule degradation in these cells (Figure 3M), showing that this residue is not critical for the ability of BtpA to protect microtubules. Together, our results show that the WxxxE motif is important for both subcellular localisation of BtpA and its ability to protect microtubules from nocodazole-induced depolymerisation.

### BtpB protects microtubules from degradation in a WxxxE-dependent manner

**Figure 4:**
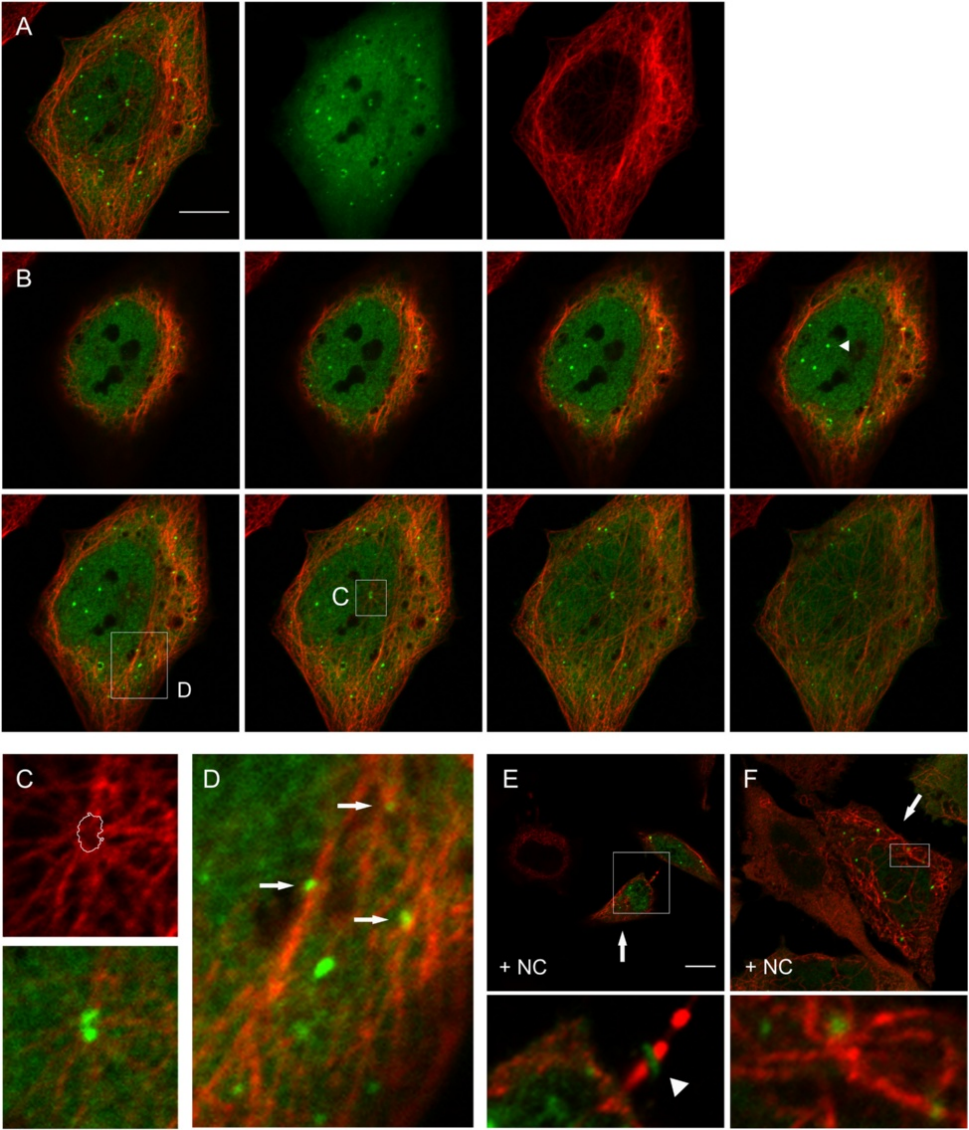
**BtpB subcellular localisation.** HeLa cells were transiently transfected with a plasmid encoding GFP-BtpB. Sixteen hours after transfection, cells were left untreated **(A-D)**, or treated **(E, F)** with 1 μg/ml nocodazole for 30 min, fixed and stained with mouse anti β-tubulin antibody, followed by detection with anti-mouse Texas Red. **(A)**. Confocal images of BtpB and the microtubule network. From left to right, merged stack images of 8 Z-slices of overlay, and individual GFP-BtpB (green) and tubulin (red) images are shown. Scale bar 10 μm. **(B)**. Images, from left to right, show the individual Z-slices (0,3 μm depth) from the cell presented in **A**. The arrow head in panel 4 indicates GFP-BtpB accumulation in the nucleus. Boxed areas in panels 4 and 6 are shown enlarged in **C** and **D**. **(C)** Individual image (red filter) showing microtubule network at the MTOC (the lines indicate the area of GFP-BtpB colocalisation, as shown in the overlay image below). **(D)** Partial association of BtpB punctae and tubulin indicated with arrows. Enlargement (10x). **(E)** and **(F)** Confocal slices of HeLa cells after nocodazole treatment (+NC). Arrows indicate individual cells showing complete protection of nocodazole-induced microtubule depolymerization in transfected cells. Boxed areas are shown enlarged. Scale bar 10 μm. **(E)** Arrow head in enlarged image points at GFP-BtpB accumulation in the Midbody of the intercellular bridge.

**Figure 5:**
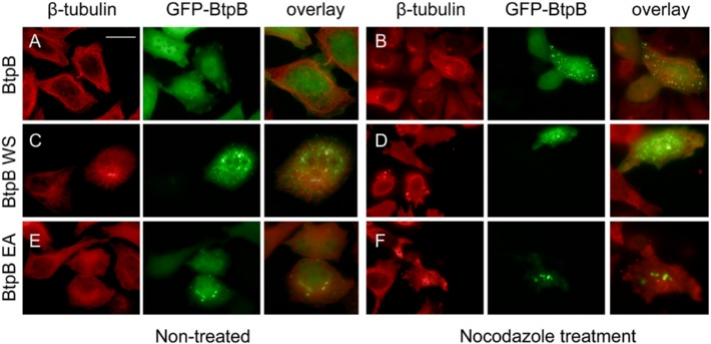
**WxxxE-dependent protection of microtubule from nocodazole-induced depolymerisation by BtpB.** HeLa cells were transiently transfected with plasmids encoding GFP-BtpB **(A-B)**, GFP-Btp W263S **(C, D)**, or GFP-BtpB E267A **(E, F)**. Sixteen hours after transfection, cells were left untreated **(A, C, E)**, or treated **(B, D, F)** with 1 μg/ml nocodazole for 30 min, fixed and stained with mouse anti β-tubulin antibody, followed by detection with anti-mouse Texas Red. **(A-F)** Individual green and red fluorescence and overlay images of HeLa cells. Scale bar, 25 μm. Data are representative of 3 or more independent experiments.

Although a role for BtpB in modulation of TLR signalling has been reported recently [17], it is not known whether BtpB associates with microtubules in a manner similar to BtpA. First, we performed ectopic expression analysis to study the subcellular localisation of BtpB. We created an N-terminal fusion of BtpB to GFP in a eukaryotic expression plasmid, and transfected HeLa cells. Using confocal and fluorescence microscopy we observed that, unlike BtpA, GFP-BtpB did not localise in filaments, but was found ubiquitously in the cytoplasm and the nucleus (Figures 4A-D, 5A), with an expression pattern varying from a diffuse GFP signal in about 50% of cells to cells which additionally showed accumulation of GFP-BtpB in one or more discrete punctae (Additional file 4: Figure S4). These discrete structures were heterogeneous in size and detected in the cytoplasm as well as the nucleus (Figure 4B). Surprisingly, in microtubule protection assays we found that BtpB was, like BtpA, able to prevent microtubules from depolymerisation by nocodazole (Figures 4E, F; 5B), suggesting that BtpB might be a microtubule-associated protein with a strong microtubule stabilizing property. Analysis of the number of transfected cells showing either complete or no protection indicated that 63.4% (±7.55, n = 110, 2 independent experiments, Table 1) showed protection of the microtubule network from depolymerisation. Full protection from nocodazole-induced destabilisation was found in a higher percentage of cells showing (at least one) BtpB puncta (82.2%), compared to protection found in cells with a diffuse BtpB signal only (24.5%) (see Table 1). This suggests that, although not crucial, the accumulation of BtpB at these visible discrete sites correlated with better protection of microtubules after nocodazole treatment.

The ability of BtpB to protect microtubules from degradation suggests that, like BtpA, BtpB might somehow associate with microtubules. Further inspection of confocal slices showed that cytoplasmic BtpB punctae showed partial colocalisation with β-tubulin (Figure 4B-D). In addition, our microscopic studies gave some further interesting observations. We often found GFP-BtpB foci at the microtubule-organizing centre (MTOC) (Figure 4C). Intriguingly, we observed that BtpB localised at a discrete ring structure in the intercellular bridge between dividing cells during late stages of cytokinesis and abscission, even in cells that had been treated with nocodazole (example shown in Figure 4E, Additional file 5: Figure S5B). Accumulated GFP-BtpB was still detected in post-mitotic midbodies (MB), associated with one of the two daughter cells after abscission, probably still connected through the thin stalk connected to the daughter cell [38].

Colocalisation studies with conjugated ubiquitin, using MAb FK2 that detects mono- and poly ubiquinated proteins, showed almost perfect colocalisation of GFP-BtpB punctae with conjugated ubiquitin, both in the cytoplasm and the nucleus. They also showed that the GFP-BtpB ring structure observed between dividing cells perfectly colocalised with FK2, demonstrating GFP-BtpB accumulates at the single, circular structure with densely ubiquitinated proteins that has been described to form in the midbody of the intercellular bridge [39] (Additional file 5: Figure S5A,B). Punctae with accumulated conjugated ubiquitin were also readily detectable in non-transfected cells (Additional file 5: Figure S5C), suggesting that in our cell transfection assays the local accumulation of conjugated ubiquitin is not dependent on BtpB overexpression. Together with the different observed subcellular localisation, these preliminary data suggest that BtpB is recruited to sites with high concentrations of ubiquitinated proteins, also during cytokinesis.

In line with our studies with BtpA, we further analysed the subcellular localisation of two GFP-BtpB WxxxE variants and their effect on the microtubule destabilising effects of nocodazole. Using site-directed mutagenesis, the substitutions W263S and E267A were introduced in the GFP-BtpB expression plasmid. Ectopic expression in HeLa cells often resulted in larger punctae than found for BtpB (Figure 5C, E). Treatment with nocodazole resulted in the formation of even larger GFP-BtpB aggregates than in non-treated cells, and these variant proteins were not able to stabilize microtubules (Figure 5D, F). These data show that also for BtpB the WxxxE motif is important for protection of microtubules.

### Ectopic expression of BtpA induces formation of filopodia in a manner that is invariably dependent of the WxxxE motif

**Figure 6:**
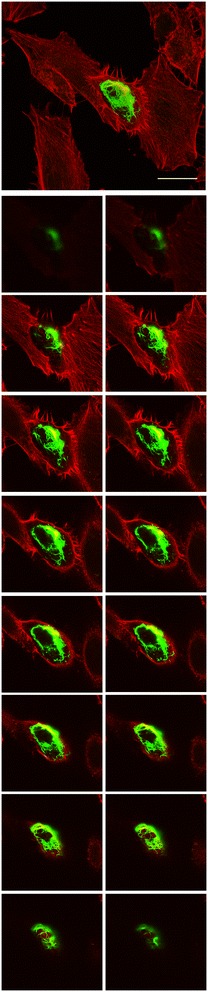
**Ectopic expression of BtpA in HeLa cells induces filopodia.** HeLa cells were transiently transfected with plasmids encoding GFP-BtpA. Sixteen hours after transfection the cells were fixed and actin was stained with Rhodamine phalloidin. Images were taken using confocal fluorescence microscopy. Large image on top, Z-Stack of 16 merged images slices (1 μm depth) of HeLa cells, showing actin distribution (red) and one cell expressing GFP-BtpA (green). The smaller images below are individual image slices. Scale bar, 25 μm. Data are representative of 3 or more independent experiments.

Despite low sequence conservation with proteins belonging to the family of bacterial WxxxE GEFs that modulate host actin dynamics, we were interested in a possible link of the Btp proteins with the actin network. Although we could not visually detect colocalisation with actin filaments in the cell for BtpA and BtpB, we detected actin rich cytoplasmic projections in HeLa cells expressing BtpA, but not BtpB, that we identified as filopodia (Figure 6). Distinctive filopodia that extend beyond the cell boundary were observed only in cells expressing GFP-BtpA, but not in non-transfected cells or the GFP control. We analysed the effect of ectopic expression of our panel of BtpA variants on the formation of filopodia. Interestingly, substitution of the conserved W and E residues, as well as G183, disrupted the ability of BtpA to induce filopodia; in contrast, the I226S variant was able to induce the formation of these actin filament bundles showing this residue is not critical for this (Table 1). These results clearly demonstrate that the conserved W213 and E217 residues are critical for the induction of filopodia in transfected HeLa cells by BtpA.

## Discussion

Intracellular bacterial pathogens employ secretion systems to introduce bacterial effectors directly into cells, where these proteins interfere with host cell biology and immune defences allowing the pathogen to survive and build an intracellular niche favourable to replication. Effectors from different pathogens have been shown to target many common cellular functions, including cytoskeleton dynamics, immune functions, GTPase signalling, host gene transcription, and ubiquitination. The stealth pathogen *Brucella* is famous for its silent entry into the host and avoidance of phagolysosomal degradation, and the translocated proteins BtpA and BtpB have been shown to contribute to this strategy by targeting the host’s TLR-dependent arm of the innate immune defence [40]. Although studies are hampered by the lack of an attenuated virulence phenotype in cell culture systems (reviewed in [9]), recently Salcedo et al., using wild type mice, suggested that BtpA and BtpB contribute to virulence and control of local inflammatory responses [17].

The molecular mechanisms by which BtpA interferes with TLR4 and TLR2 signalling pathways remain controversial, due to the inconsistency between reports describing interaction with the adaptor proteins MyD88 and/or TIRAP/MAL [19,24,26,27,41]. Recent structural studies are now complementing biochemical, *in vitro* and overexpression studies, and the picture starts to emerge that BtpA may inhibit downstream MyD88-dependent signalling by competing with TIRAP in binding to the TLR4 receptor protein [25,28,29]. This process has been suggested to take place at the plasma membrane, possibly through a phosphoinositide interaction module with which BtpA inserts itself in the membrane [23-25]. However, the situation seems more complex, as BtpA has also been shown to be involved in microtubule dynamics and, very recently, in the induction of the unfolded protein response [30,42].

Our data corroborated previous reports showing that BtpA interacts with microtubules *in vitro* [30], and that ectopically expressed BtpA formed a filamentous network in HeLa cells that partially colocalised with microtubules [24]. We found that this association was essential to block drug-induced depolymerisation of microtubules.

The second *Brucella* TIR protein, BtpB, has only recently been demonstrated to play a role in interference with TLR signalling [17], and the molecular mechanism of interference is as yet unknown. We were interested to find out whether BtpB is able to associate with the microtubule network. Here, we provide evidence that ectopically expressed BtpB equally protects microtubules from nocodazole-induced degradation in a WxxxE dependent manner. This result places BtpB at the interface of the microtubule network and TLR signalling, similar to BtpA. We observed partial colocalisation of visible BtpB punctae with β-tubulin in confocal slices, and the presence of BtpB punctae was correlated with better protection against microtubule depolymerisation. Our results further showed that BtpB colocalises with sites of accumulated ubiquitinated proteins, which were equally observed in non-transfected cells, not only in the cytoplasm but also in the nucleus and at a discrete region in the central spindle region between dividing cells, to which cytokinetic proteins are recruited to promote cell division. The central spindle is a bundle with interdigitated antiparallel microtubules between separating chromosomes. The FK2-positive Midbody ring has been shown to be a dynamic site of ubiquitination during the important later stages of cell division and abscission, when the cells finally detach [39]. More experiments are needed to determine if it is an association with microtubules or the UPS that causes the accumulation of ectopically expressed GFP-BtpB at the Midbody ring during cell division. Although colocalisation of BtpB punctae with conjugated ubiquitin raises the question as to whether BtpB itself could be sequestered by one of the cellular host mechanisms that deals with misfolded proteins [43-45], our preliminary observations provide an indication for a functional link of BtpB, microtubules and the Ubiquitin Proteasome system (UPS). Many viral and bacterial proteins have been shown to interfere with host ubiquitination processes [46,47], and further experiments should provide more information whether BtpB could for instance enhance poly ubiquitination of TIR proteins, similar as shown for BtpA which targets the TIR adaptor TIRAP for degradation by polyubiquination [27].

Modulation of the host cytoskeleton by bacterial virulence factors is a common microbial strategy used by many pathogens at different stages of the infection process (reviewed in [48]. The actin network is often modulated by bacterial proteins that target the host small GTPases that are the essential signalling intermediates in actin dynamics [49]. During our studies we found that, besides colocalising with microtubules, ectopically expressed BtpA, but not BtpB, also induced actin rich filopodial protrusions in HeLa cells, a mainly Cdc42-dependent process. A WxxxE motif, that we identified in BtpA and BtpB led us to hypothesise that BtpA may induce GTPase activation in a similar manner as proteins belonging to the family of WxxxE/SopE T3SS effectors, most of which have been demonstrated to be Rho-GEFs. Ectopic expression of these WxxxE effectors has been shown to result in WxxxE-dependent formation of filopodia, stress fibers or lamellipodia, phenotypes that are typically associated with selective activation of the Rho GTPases Cdc42, RhoA and Rac1, respectively [34,50]. Our expression studies in HeLa cells revealed that conservation of the W and E residues was crucial for the ability of BtpA to induce actin rearrangements, similar to the WxxxE GEF family effectors [32,51,52]. Although BtpA did not show sequence conservation with the different WxxxE GEF family proteins, the induction of filopodia in a WxxxE-dependent manner raised the question of whether BtpA could also function as a Cdc42 GEF, similar to the EPEC Map protein, that induces filopodia around the infecting bacterium [34,50]. Despite many attempts, including direct RhoGEF exchange assays, we have, however, been unable to unambiguously show activation of Cdc42 by BtpA (not shown). To our knowledge, formation of functional filopodia has never been reported during *Brucella* infection; however, the ectopic induction of filopodia could be a consequence of activation of GTPases other than Cdc42, and play a localised intracellular role during *Brucella* infection. How ectopically expressed BtpA is able to induce these specific ectopically induced actin protrusions remains a subject for future studies.

We further analysed a role for the WxxxE motif in microtubule dynamics. Our mutagenesis studies revealed that the motif was not only required for induction of filopodia, but also for interaction with microtubules. Changes of both the Trp and the Glu residue affected BtpA localisation. As expected, proper subcellular localisation in tubular structures (albeit with different efficiencies for the individual variants) was required for microtubule protection for all analysed variants (Table 1). Changes to the Trp residue only affected the subcellular localisation efficiency but did not alter the ability of BtpA to protect microtubules once at the proper localization (W213F). Interestingly, however, negative charge at the Glu position seemed criticial for protection (the E217A variant was localised in tubules in about 53% of the transfected cells, yet could not protect microtubules). In addition, the motif was also important for the ability of BtpB to protect microtubules from degradation. Thus the WxxxE motif is important for microtubule protection by both BtpA and BtpB with, and negative charge in the WxxxE pocket may be crucial for this.

In the family of WxxxE GEFs, the conserved WxxxE motif has been shown to position a catalytic loop, that is essential for interaction with, and selective activation of specific Rho GTPase isoforms, exemplified by the interaction between Map and the β2-3 interswitch region of Cdc42 [31,34]. Our recent resolution of the crystal structure of the BtpA TIR domain [28] shows that the WxxxE motif, located just adjacent of the αC helix in TIR box 2, positions the BB loop through interaction with a D residue (Figure 2B). Our mutagenesis results indicate that, although the motif is important for both colocalisation of BtpA with microtubules as well as its ability to protect microtubules from drug-induced degradation, minor differences in hydrophobic interaction in the WxxxE pocket between the different variants, may result in differences in positioning of the BB loop with either flexible (E217A) or stable conformations (E217D). This again may result in the observed differences in efficiency of colocalisation. Interestingly, this conformation positions the G183 residue that has been shown to be critical for interaction of BtpA with microtubules ([30] and this study), and shows that the WxxxE motif is an important structural motif in the interaction of BtpA with the microtubule network.

Extensive phylogenetic analysis identified 1688 TIR containing proteins, including 483 from both pathogenic and non-pathogenic bacteria [35]. We found that a WxxxE motif is present in a highly conserved region of almost all bacterial TIR proteins including the well-studied TcpC, PdTLP, SaTLP and TlpA [53], but also in eukaryotic TLR proteins TLR1, 2, 4, 6, and 10 and TIR adaptor protein SARM. Whereas positioning of a catalytic loop involved in interaction with GTPases has been described for members of the family of WxxxE GEFs, in bacterial TIR proteins the WxxxE motif may be involved in positioning residues important for association with microtubules, as shown for BtpA, or in positioning different loop structures.

Summarizing, our functional and structural studies show that the *Brucella* Btp proteins have a structural WxxxE motif important for their function. For several reasons, including little overall sequence conservation of BtpA and BtpB with the WxxxE/SopE family GEFs, the fact that BtpB does not induce any detectable actin rearrangements, and the conservation of the WxxxE motif in a large family of both pro- and eukaryotic TIR proteins, BtpA and BtpB are likely not part of the WxxxE family of GEFs. Recently, the *Bartonella* type IV secretion effector BepF, which has been shown to be involved in activation of Rac1 and Cdc42 in a WxxxE-dependent manner, has been suggested also likely not to represents a WxxxE/GEF family member [54]. This raises the question of whether there is an evolutionary advantage of the WxxxE motif as a strong structural determinant which may position important functional domains or catalytic sites. Further studies, however, are needed to define a possible role for the motif in other TIR domain containing proteins before conclusions can be drawn about the existence of other WxxxE families. Based on the WxxxE-dependent ability of ectopically expressed BtpA to induce filopodia, we propose that BtpA may help gain more insight into the frequently described cross talk between TLR and GTPase signalling networks [55,56].

The precise role of BtpA and BtpB during *Brucella* infection remains unknown. Both proteins interfere with TLR signalling and somehow associate with microtubules, but they seem to be independently acquired functions. We believe that the GIs carrying *btpA* and *btpB* were acquired when the ‘classical’ *Brucella* separated from the older species (*B. inopinata*, Australian rodent strains) and played a role in the development of this bacterium’s ‘stealth’ approach. The distribution of the GI carrying *btpA* suggests that it was lost through independent excision events several times in the evolution of the genus (Additional file 1: Figure S1). Whereas BtpB is present in all sequenced classical *Brucella* species, BtpA is absent in for instance *B. suis*. Interestingly, in all *B. melitensis* the tryptophan of the WxxxE motif has been changed into arginine. It remains to be analysed whether this rendered the protein non-functional, or whether it acquired other functions.

Interestingly, in dendritic cells, intracellular TLR2 and TLR4 have been shown to associate with microtubules and the Golgi, and this interaction was essential for IL-12 production in response to the intracellular pathogen *Neisseria meningitides* [57]. This situation seems reminiscent of the interaction of BtpA with TLR4 [25] and microtubules. We hypothesize that an intracellular TLR4 signalling complex is formed in endosomes that requires the microtubule network, and that BtpA interferes with locally induced signalling. Our findings that BtpB has a similar WxxxE-dependent microtubule protective role, but does not result in the induction of filopodia after ectopic expression, further suggests these two *Brucella* virulence proteins may play partially overlapping, yet different roles in virulence.

## Materials and methods

### Bacterial strains

For cloning *Escherichia coli* DH5α was cultured at 37°C in Luria-Bertani broth (LB) (Invitrogen, Merck). Kanamycin (25 μg/ml) or chloramphenicol (30 μg/ml) were added to the media when appropriate.

### Plasmids and site-directed mutagenesis

A 828 bp version of the *btpA* gene was cloned from *B. melitensis* 16M genomic DNA (BMEI1674, Genbank accession NP_540591 is the shorter annotated version) using PCR (primers *Bgl*II L1674-F 5’-GA*AGATCT*TATGAGTTCGTACTCTTCTAATATTG-3’, and *Pst*IL1674-R 5’-AA*CTGCAG*TCAGATAAGGGAATGCAGTTC-3’) and cloned in frame with the GFP coding sequence in eukaryotic expression plasmid pEGFP (Clontech) using standard protocols, resulting in plasmid pIN271.

A 978 version of the *btpB* gene was cloned from *B. suis* genomic DNA (BR0735, Genbank accession number AAN29664 is the shorter annotated version) using PCR (primers *Bgl*II 735-F 5’-GA*AGATCT*TATGACATCTAGTCGCGACACG-3’, and *Pst*I 735-R 5’-AA*CTGCAG*CTAGGTGATGAGGGCGACG-3’) and cloned in frame with the GFP coding sequence in pEGFP resulting in plasmid pIN292. We amplified a longer version of *btpB*, which was recently also shown to complement a *B. abortus btpB* mutant in the control of NF-κB translocation into the nucleus [17].

Site directed mutagenesis of the WxxxE motif was done using the QuickChange Site-Directed Mutagenesis kit (Stratagene) following manufacturer’s instructions, using pIN271 as a template with the following mutagenic primers for *btpA* (changed codons for Trp (TGG) and Glu (GAA) are italicised): W213A (5’-TTTAGCAAGCAA*GCG*CCCGCAAGAGAATTAG-3’), W213S (5’-TTTAGCAAGCAA*TCG*CCCGCAAGAGAATTAG-3’), W213F (5’-TTTAGCAAGCAA*TTC*CCCGCAAGAGAATTAG-3’), E217A (5’-CAATGGCCCGCAAGA*GCA*TTAGATGGACTGAC-3’), E217D (5’- CAATGGCCCGCAAGA*GAT*TTAGATGGACTGAC-3’), I226S (5’- CTGACGGCAATGGAA*AGT*GGCGGACAGACGC-3’), G183A (5’- CATATACGTTGAAGGTC*GCT*GACAGCCTTCGGCG-3’), and *btpB* (pIN292 as a template): W263S (5’- CTATCAGCGAAAAGAC*TCG*TGCGGCGTCG-3’) and E267A (5’- CTGGTGCGGCGTC*GCG*TTCCGCGCGATTCG-3’). Constructs were verified by DNA sequencing (Eurofins MWG operon, Germany). Table 2 summarizes the plasmids used in this study.

**Table 2:** **Strains and plasmids used in this study**

**Strain or plasmid**	**Relevant characteristics**	**Source**
*E. coli* DH5α	DH5α F − 80*lacZ*Δ*M15* Δ(*lacZYA-argF*)*U169 endA1 recA1 hsdR17* (rK − mK+) *supE44 thi-1*Δ*gyrA96 relA1*	
pEGFP-C3	plasmid containing an enhanced GFP expression unit under control of the CMV promoter, Km^r^	Clontech
pIN271	pEGFP derived plasmid containing GFP-*btpA* expression unit, Km^r^	This Study
pIN275	pIN271-derivative carrying *btpA* W213A	This Study
pIN269	pIN271-derivative carrying *btpA* W213S	This Study
pIN274	pIN271-derivative carrying *btpA* W213F	This Study
pIN270	pIN271-derivative carrying *btpA* E217A	This Study
pIN277	pIN271-derivative carrying *btpA* E217D	This Study
pIN262	pIN271-derivative carrying *btpA* I262S	This Study
pIN283	pIN271-derivative carrying *btpA* G183A	This Study
pIN292	pIN271-derivative carrying *btpB*	This Study
pIN293	pIN271-derivative carrying *btpB* W263S	This Study
pIN294	pIN271-derivative carrying *btpB* E267A	This Study

### Cell culture, transfection, immunocytochemistry, and fluorescence microscopy

HeLa cells were grown in RPMI 1640 (Gibco) supplemented with heat-inactivated 10% fetal bovine serum (FBS, Lonza, Switzerland). Transfections were performed using Lipofectamine 2000 (Invitrogen). For immuno fluorescence studies, cells were seeded on coverslips (BD Bioscience) and cultured overnight in 12-well dishes. Cells were transfected and, after 16–20 hours, treated with nocodazole (Sigma M1404) at a concentration of 1 μg/ml for 30 min if desired. Cells were fixed with 4% PFA, and processed for immunocytochemistry. Monoclonal mouse anti-β-tubulin (Sigma Aldrich, T4026), mouse anti-FK2 (Enzo Life science, BML-PW8810), Texas Red anti-mouse (Vector Laboratories, TI-2000), Rhodamin phalloidin (Invitrogen R415), Rabbit anti-BtpA (a gift from Marty Roop, East Carolina University, Greenville), FITC anti-rabbit (Vector Laboratories, FI-2000) were used for immune labelling. Immuno fluorescence microscopy was performed using a LEICA DM/IRB microscope using filter sets L5 (band pass (BP) 480/40; Beam splitter (BS) 505; emission BP527/30) and N2.1 (515–560; BS 580; emission long pass (LP) 590), respectively. For imaging we used a Coolsnap fx (Roper Scientifique) and MetaVue software, and images were further processed using Adobe Photoshop. Confocal analysis was performed at the RIO imaging platform in Montpellier, with a Biorad MRC1024 confocal microscope.

### Bio informatic analysis

*Brucella* genomes were obtained and compared on the PATRIC website [33]. Sequence alignments were performed using T-Coffee analysis, and further analysed using Jalview. Structural figures were generated with Pymol (www.pymol.org).

## Additional files

Additional file 1: Figure S1. Evolutionary tree of *Brucella* species and distribution of BtpA and BtpB proteins. The alignment was made in Patric and the tree was optimized using Mega Software. *btpA* and *btpB* genes are highly conserved throughout the genus. We found only four variations in 407 sequences (showing 47 species). All *B. melitensis* strains carry a single amino acid change (leading to A163V), while three strains carry a variation in R167H (*B. ovis*), G200D (*B. suis* ATCC23445) or V182G (*B. neotomae*). BtpB is also highly conserved, with only two amino acids that show variation. The Trp residue, as part of a WxxxE motif, in BtpB was replaced by an Arg residue in *B. melitensis* BtpB (W263R). Additional file 2: Figure S2. Confocal imaging of BtpA in HeLa cells shows tubular localisation at cell periphery. HeLa cells were transiently transfected with a plasmid encoding GFP-BtpA, fixed after 16 h and analysed by confocal microscopy. The coloured image (green) shows the merged stack images of 14 Z-slices, the other images show the individual Z-images (1 μm depth) of HeLa cells expressing GFP-BtpA. Bar, 25 μm. Additional file 3: Figure S3. Ectopic expression of BtpA in HeLa cells shows colocalisation with microtubules HeLa cells were transiently transfected with a plasmid expressing GFP-BtpA. At 16 h cells were fixed and processed for immuno labelling with mouse anti β-tubulin antibody, detected with anti-mouse Texas Red to visualize microtubules (red fluorescence). The large image shows a merged image of 6 Z-Stack confocal images (1 μm depth) of cells with microtubules (in red) and GFP-BtpA in one of the cells. Arrow heads indicate colocalisation of GFP-BtpA with the microtubule network (yellow), at the periphery of the cell. The other images represent the individual Z-stack images. Scale bar, 25 μm. Data are representative of 3 or more independent experiments. Additional file 4: Figure S4. Expression phenotypes of GFP-BtpB in HeLa cells. HeLa cells were transiently transfected with a plasmid expressing GFP-BtpB. At 16 h cells were fixed, processed, and analysed using fluorescence microscopy. The individual images show the different observed expression patterns ranging from a diffuse signal, to cells with a diffuse fluorescent signal as well as accumulation of BtpB in punctae, heterogeneous in size and number. Scale bar, 25 μm. Additional file 5: Figure S5. Colocalisation of BtpB and conjugated ubiquitin. HeLa cells were transiently transfected with a plasmid expressing GFP-BtpB. At 16 h cells were fixed and processed for immune labelling with mAb FK2, which detects mono- and poly conjugated ubiquitin, and labelled with anti-mouse Texas Red for fluorescence detection. A. Confocal images showing stacks of overlay and individual GFP-BtpB and FK2 images. Scale bar, 10 μm. B. Two fluorescence images (red and green filters) showing colocalisation of BtpB and FK2, also at the intercellular bridge in late cytokinesis (arrow, and boxed areas). Scale bar, 25 μm C. Image showing transfected and non-transfected cells with and without FK2 positive foci. Cells indicated with numbers in the overlay panel (on the right) are enlarged below as individual green and red fluorescent images. Scale bar, 25 μm. 1, 2. Transfected cells with a punctate pattern for BtpB, colocalising with FK2. FK2 positive, BtpB negative foci are also observed. 3, 4: Non-transfected, FK2 positive foci. 5. Transfected, no visible BtpB punctae, FK2 positive.

## Abbreviations

GEF: Guanidine exchange factors
LPS: Lipopolysaccharide
MTOC: Microtubule-organizing centre
NC: Nocodazole
TIR: Toll/Interleukin-1 receptor
TLR: Toll like receptor
T4SS: Type IV secretion system
T3SS: Type III secretion system
UPS: Ubiquitin Proteasome system
