# Supplementary figures and images for: The Brucella TIR domain containing proteins BtpA and BtpB have a structural WxxxE motif important for protection against microtubule depolymerisation
Source: Cell Commun Signal. 2014 Oct 12;12:53. doi: 10.1186/s12964-014-0053-y (PMC4203976; doi:10.1186/s12964-014-0053-y)

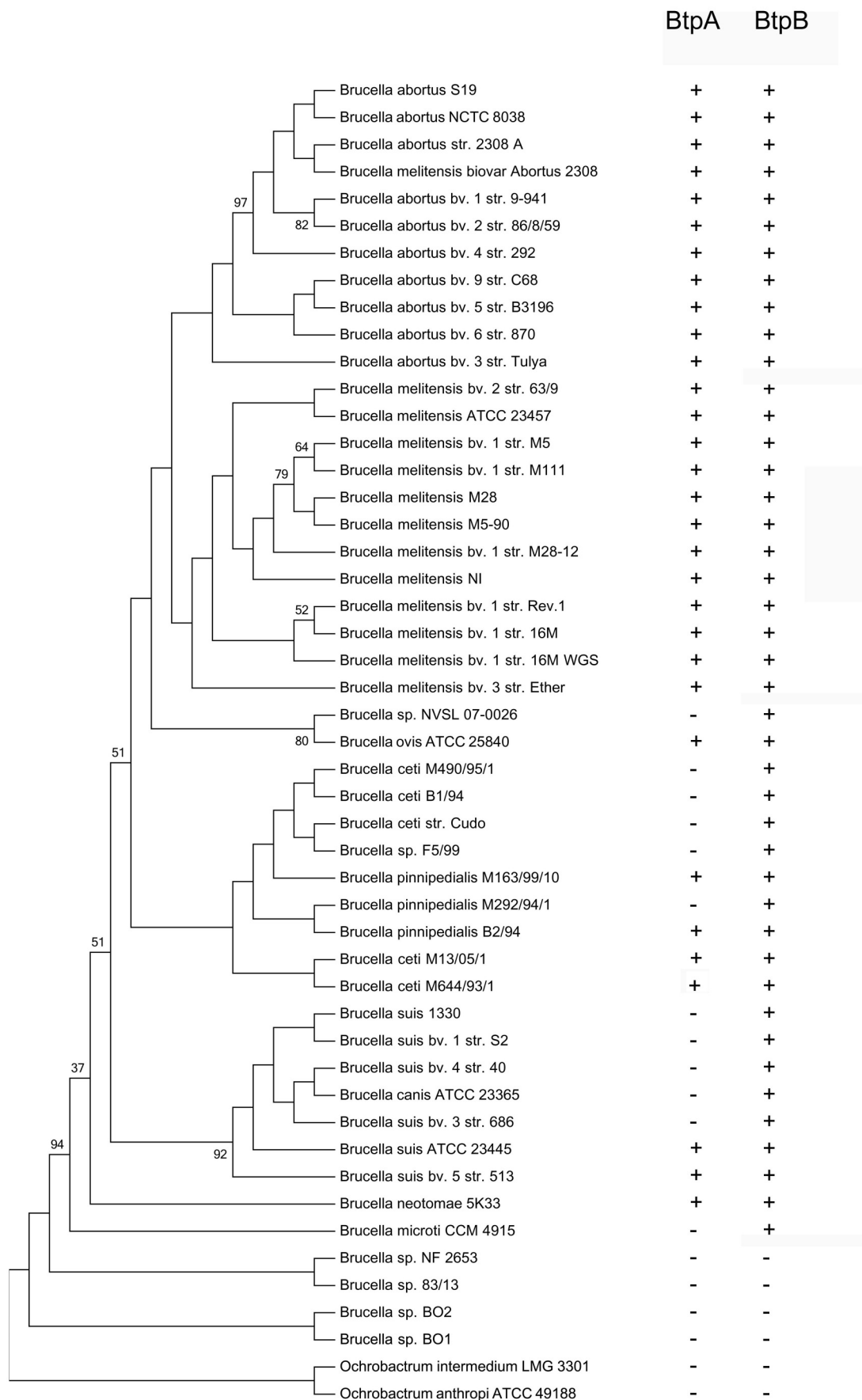

**Figure S1**

Supplement: Additional file 1: Figure S1. — Evolutionary tree of Brucella species and distribution of BtpA and BtpB proteins. The alignment was made in Patric and the tree was optimized using Mega Software. btpA and btpB genes are highly conserved throughout the genus. We found only four variations in 407 sequences (showing 47 species). All B. melitensis strains carry a single amino acid change (leading to A163V), while three strains carry a variation in R167H (B. ovis), G200D (B. suis ATCC23445) or V182G (B. neotomae). BtpB is also highly conserved, with only two amino acids that show variation. The Trp residue, as part of a WxxxE motif, in BtpB was replaced by an Arg residue in B. melitensis BtpB (W263R). [file 12964_2014_53_MOESM1_ESM.pdf]

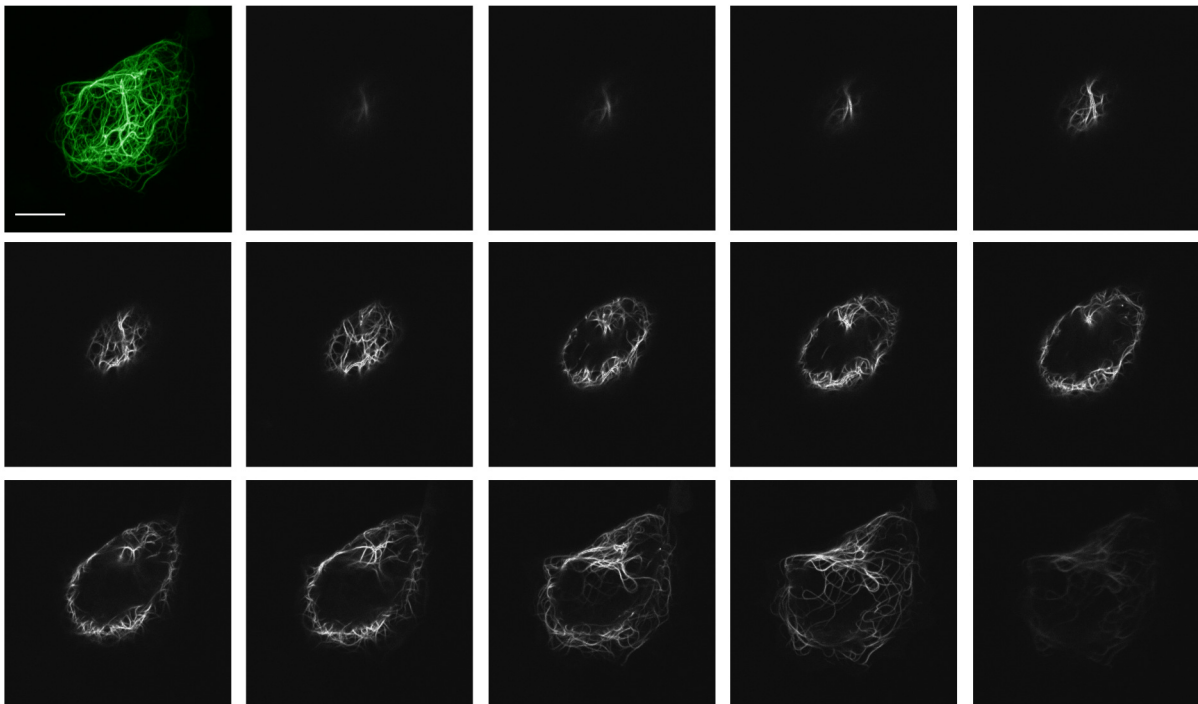

**Figure S2**

Supplement: Additional file 2: Figure S2. — Confocal imaging of BtpA in HeLa cells shows tubular localisation at cell periphery. HeLa cells were transiently transfected with a plasmid encoding GFP-BtpA, fixed after 16 h and analysed by confocal microscopy. The coloured image (green) shows the merged stack images of 14 Z-slices, the other images show the individual Z-images (1 μm depth) of HeLa cells expressing GFP-BtpA. Bar, 25 μm. [file 12964_2014_53_MOESM2_ESM.pdf]

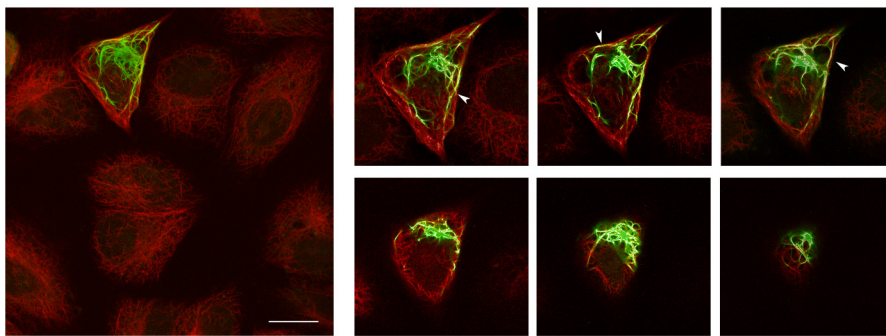

**Figure S3**

Supplement: Additional file 3: Figure S3. — Ectopic expression of BtpA in HeLa cells shows colocalisation with microtubules HeLa cells were transiently transfected with a plasmid expressing GFP-BtpA. At 16 h cells were fixed and processed for immuno labelling with mouse anti β-tubulin antibody, detected with anti-mouse Texas Red to visualize microtubules (red fluorescence). The large image shows a merged image of 6 Z-Stack confocal images (1 μm depth) of cells with microtubules (in red) and GFP-BtpA in one of the cells. Arrow heads indicate colocalisation of GFP-BtpA with the microtubule network (yellow), at the periphery of the cell. The other images represent the individual Z-stack images. Scale bar, 25 μm. Data are representative of 3 or more independent experiments. [file 12964_2014_53_MOESM3_ESM.pdf]

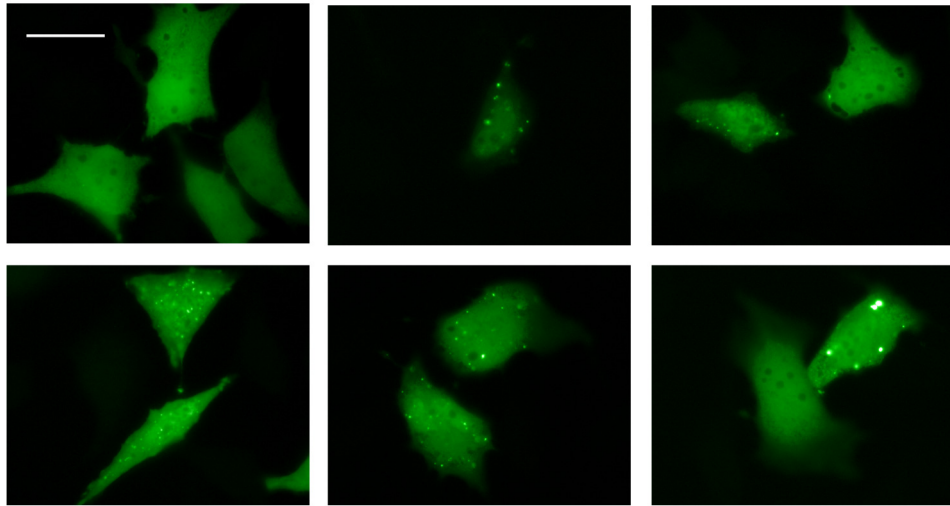

**Figure S4**

Supplement: Additional file 4: Figure S4. — Expression phenotypes of GFP-BtpB in HeLa cells. HeLa cells were transiently transfected with a plasmid expressing GFP-BtpB. At 16 h cells were fixed, processed, and analysed using fluorescence microscopy. The individual images show the different observed expression patterns ranging from a diffuse signal, to cells with a diffuse fluorescent signal as well as accumulation of BtpB in punctae, heterogeneous in size and number. Scale bar, 25 μm. [file 12964_2014_53_MOESM4_ESM.pdf]

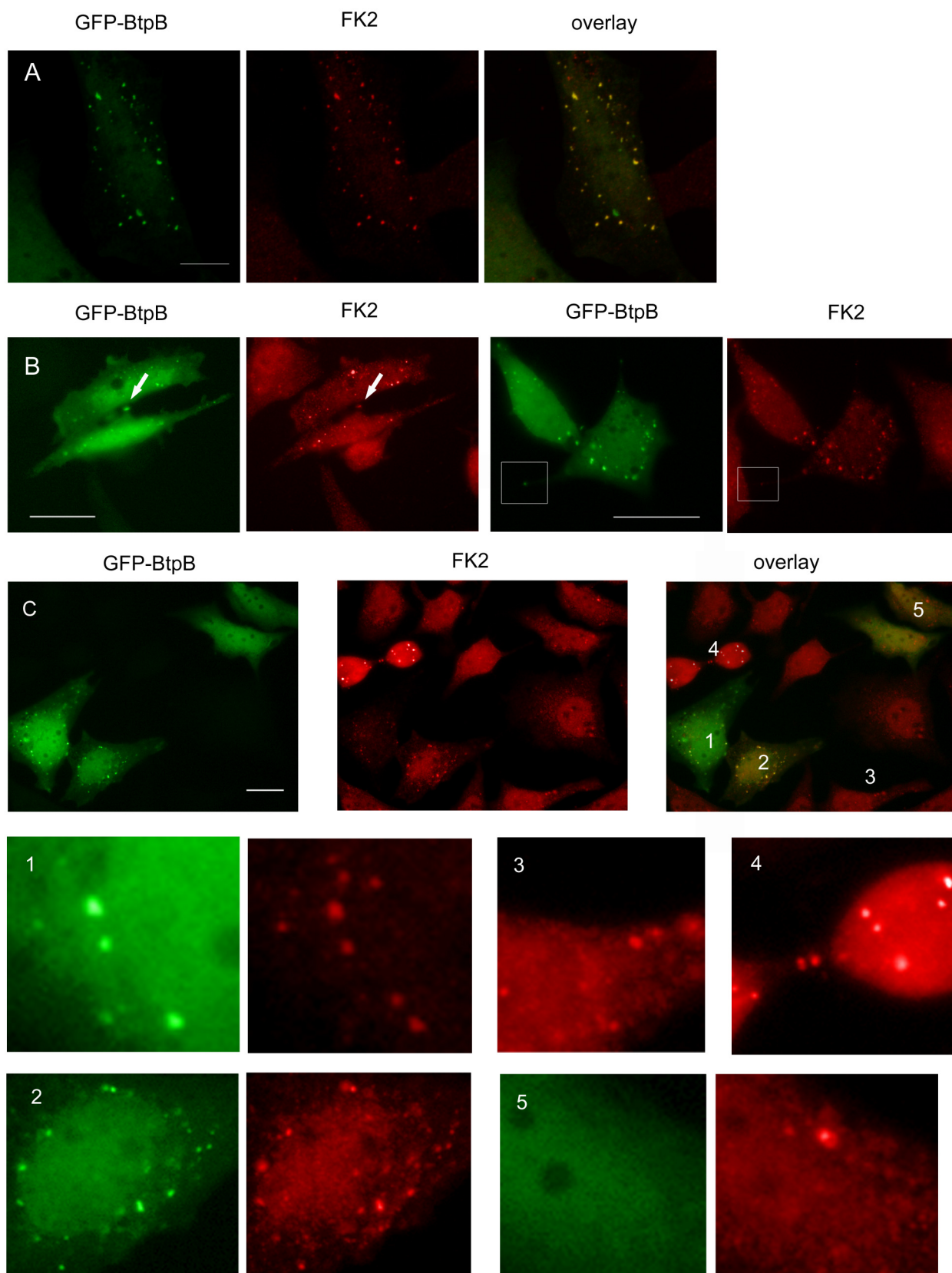

**Figure S5**

Supplement: Additional file 5: Figure S5. — Colocalisation of BtpB and conjugated ubiquitin. HeLa cells were transiently transfected with a plasmid expressing GFP-BtpB. At 16 h cells were fixed and processed for immune labelling with mAb FK2, which detects mono- and poly conjugated ubiquitin, and labelled with anti-mouse Texas Red for fluorescence detection. A. Confocal images showing stacks of overlay and individual GFP-BtpB and FK2 images. Scale bar, 10 μm. B. Two fluorescence images (red and green filters) showing colocalisation of BtpB and FK2, also at the intercellular bridge in late cytokinesis (arrow, and boxed areas). Scale bar, 25 μm C. Image showing transfected and non-transfected cells with and without FK2 positive foci. Cells indicated with numbers in the overlay panel (on the right) are enlarged below as individual green and red fluorescent images. Scale bar, 25 μm. 1, 2. Transfected cells with a punctate pattern for BtpB, colocalising with FK2. FK2 positive, BtpB negative foci are also observed. 3, 4: Non-transfected, FK2 positive foci. 5. Transfected, no visible BtpB punctae, FK2 positive. [file 12964_2014_53_MOESM5_ESM.pdf]
